# The Sleep Condition Indicator and the Idiopathic Hypersomnia Severity Scale: Measurement Invariance and an Exploratory Network Approach in a French Sample of University Students

**DOI:** 10.1111/jsr.70042

**Published:** 2025-04-10

**Authors:** Sophie Bayard, Julie Faccini, Jonathan Del‐Monte, Clarisse Madiouni

**Affiliations:** ^1^ Université Paul Valéry Montpellier 3 University of Montpellier Montpellier France; ^2^ Laboratory of Clinical, Cognitive and Social Anthropology and Psychology (LAPCOS) University of Cote d'Azur Nice France; ^3^ Emotions, Neurocognition and Therapeutic Behavioral Approaches (ENACT) Team University of Nîmes Nice France

**Keywords:** hypersomnolence, insomnia, network analysis, structural invariance, university students

## Abstract

Insomnia and hypersomnolence symptoms are prevalent among university students, yet their assessment methods face limitations, and the relationship between these symptoms remains underexplored. We examined the structural invariance of the Sleep Condition Indicator (SCI) and the Idiopathic Hypersomnia Severity Scale (IHSS) in university students. In addition, we proposed a network approach to the insomnia and hypersomnolence symptoms. A total of 433 university students underwent a clinical interview for sleep and socio‐demographics and completed the SCI and the IHSS. Confirmatory factorial and network analyses were conducted. The SCI demonstrated a two‐factor structure, while the IHSS exhibited a three‐factor structure. Over 70% of students scored above the IHSS clinical threshold, while 36.5% were diagnosed with insomnia. For the SCI, a threshold of ≤ 13 demonstrated the highest predictive value for diagnosing insomnia. Network analyses highlighted the central role of cognitive consequences of insomnia and hypersomnolence. Cognitive and emotional consequences of insomnia and hypersomnolence were moderately interconnected. Higher cognitive complaints related to insomnia were associated with increased feelings of insufficient sleep and more pronounced sleep inertia. Additionally, greater wakefulness after sleep onset was linked to both a shorter ideal night‐time sleep duration and increased difficulty staying awake during low‐stimulation activities throughout the day. The SCI and IHSS showed structural invariance in university students when compared to the general population. Insomnia and hypersomnolence represent critical clinical issues among French students. We underscored the intricate relationship between insomnia and hypersomnolence, highlighting the urgent need for targeted interventions that address both daytime and nighttime sleep–wake disturbances.

## 1 Introduction

More than two‐thirds of university students present self‐reported symptoms suggestive of sleep disorders, such as insomnia, hypersomnolence and circadian rhythm disorders (Yassin et al. 2020). More specifically, up to more than half of all university students can experience symptoms of insomnia, including poor sleep quality, short sleep duration and irregular sleep schedules (Babicki et al. 2023; Binjabr et al. 2023; Carrión‐Pantoja et al. 2022; Jiang et al. 2015). More than a third of university students experience clinically significant levels of excessive daytime sleepiness, alongside insomnia (Babicki et al. 2023; Bjørnnes et al. 2021). In this context, sleep–wake disturbances are related to poor academic achievement and poor mental health status (Carrión‐Pantoja et al. 2022; Seoane et al. 2020). Therefore, addressing sleep‐related challenges among university students is essential for supporting their well‐being and academic achievement.

The Pittsburgh Sleep Quality Index (PSQI) (Buysse et al. 1989), the Insomnia Severity Index (ISI) (Morin 1993) and the Epworth Sleepiness Scale (ESS) (Johns 1991) are the three main tools used to assess insomnia and hypersomnolence constructs in university students (Babicki et al. 2023; Carrión‐Pantoja et al. 2022; Dietch et al. 2016; Emert et al. 2024; Seoane et al. 2020). Although the PSQI offers valuable methodological features for screening sleep disturbances, it is not specific to insomnia (Ali et al. 2020). Furthermore, the structural invariance of the ISI (seven‐item), varying from one to three factors, is not clearly established in the literature (Ali et al. 2020; Lenderking et al. 2024). This issue also applies to the ESS (Gonçalves et al. 2023). Structural invariance in psychometrics means that the structure of a psychological construct stays consistent across different groups or conditions. This ensures that measurement models, like the factor structure of a questionnaire, are comparable across subgroups (e.g., gender, age or culture). It is crucial for valid cross‐group comparisons, as it confirms that differences are due to the construct itself, not measurement inconsistencies. Another issue concerns the fact that the ESS exclusively addresses one symptom of hypersomnolence: daytime sleepiness. As a result, in this field of research, neither sleep inertia nor excessive sleep duration (at night or during the day) is typically included in the hypersomnolence construct as defined in the ICSD‐3 and the DSM‐5 (American Academy of Sleep Medicine 2014; American Psychiatric Association 2013). In this context, the interrelationships between insomnia and hypersomnolence symptoms as defined by the ICSD‐3 and the DSM‐5 remain currently unexplored in university students. Finally, it should be noted that the Sleep Condition Indicator (SCI) and the Idiopathic Hypersomnia Severity Scale (IHSS) are two well‐validated scales in the literature that respectively assess insomnia and hypersomnolence symptoms according to DSM‐5 and ICSD‐3 classifications. Both tools have shown structural invariance, in general, populations (Bayard et al. 2017; Dauvilliers et al. 2019; Espie et al. 2014; Madiouni et al. 2024).

The objectives of the current study were threefold. First, we assessed whether the SCI and IHSS maintain structural invariance in university students. Second, we evaluated the applicability of their clinical thresholds in this specific population. Finally, we used an exploratory network approach to explore how insomnia and hypersomnolence symptoms interact. By achieving these objectives, we aimed to enhance the reliability of using the SCI and IHSS in university student populations. The network analysis offers a valuable complement to traditional methods such as structural equations, particularly for exploring the complexity of insomnia and hypersomnolence symptoms interactions. Unlike traditional models, the network approach does not assume linear or predetermined relationships between latent and observed variables (Briganti et al. 2024). Instead, it provides a direct visualisation and analysis of insomnia and hypersomnolence symptoms interconnections without prior assumptions about their structure. This approach allows for a more natural exploration of how symptoms coexist and interact. Furthermore, network analysis identifies the most influential insomnia and hypersomnolence symptoms within the overall network, helping to target interventions more effectively by focusing on the most interconnected symptoms.

## 2 Methods

### 2.1 Participants

Four hundred and thirty‐three university students were recruited from universities in the Occitanie, Nouvelle‐Aquitaine and Provence‐Alpes‐Côte d'Azur regions of France. The eligibility criteria included an age of 18 years or older, to be French speakers and to be enrolled in a bachelor's or master's degree. Students were excluded if they had a history of schizophrenia spectrum or other psychotic disorders, bipolar‐related disorders, neurological diseases including cerebrovascular diseases and traumatic brain injuries or an active, progressive or unstable physical illness (e.g., cancer or acute pain). Also, pregnant women, breastfeeding mothers and parents with a baby < 12 months old were excluded.

### 2.2 Clinical Interview and Questionnaires

During the face‐to‐face clinical interview, we assessed insomnia disorder based on the DSM‐5 and ICSD‐3 criteria (American Academy of Sleep Medicine 2014; American Psychiatric Association 2013), chronic diseases, psychotropic medication and socio‐demographics, including the degree being pursued and the financial status. Insomnia disorder was diagnosed using a local translation of the clinical version of the structured interview for DSM‐5 (SCID‐5‐CV). Insomnia disorder was diagnosed in students if they (1) self‐reported difficulty in initiating and/or maintaining sleep and/or early morning awakening despite having adequate opportunity to sleep, (2) were undergoing significant distress and/or impairment in daytime functioning and (3) the symptoms occurred at least three nights a week for at least 3 months, according to the SCID‐5‐CV criteria. All participants were screened for restless legs syndrome/Willis‐Ekbom (RLS/WED) disease and for obstructive sleep apnea. RLS/WED was assessed based on the International Restless Legs Syndrome Study Group criteria. The Berlin questionnaire was applied to screen possible obstructive sleep apnea. It consists of three categories of questions related to snoring, daytime sleepiness and fatigue and high blood pressure/body mass index. There is a risk of obstructive sleep apnea if the participant has two out of the three positive categories. Participants were asked about a self‐reported lifetime diagnosis of central hypersomnia.

#### 2.2.1 IHSS

The IHSS is a 14‐item scale, evaluating the severity, frequency and functional impact of idiopathic hypersomnia symptoms. Responses are rated on a 4‐point Likert scale. Total scores range from 0 to 50. A total score greater than or equal to 22 is the recommended cutoff value for discriminating between untreated patients with idiopathic hypersomnia and controls (Dauvilliers et al. 2019). Severity categories based on the total score are proposed: *mild* (0–12), *moderate* (13–25), *severe* (26–38) and *very severe* (39–50) (Rassu et al. 2022). In the general population, the IHSS is characterised by three factors: Nighttime sleep/inertia (Items 1, 2, 3, 4, 5 and 8), daytime sleepiness (Items 6, 7 and 9) and daytime consequences (Items 10, 11, 12, 13 and 14) (Madiouni et al. 2024).

#### 2.2.2 SCI

SCI is an eight‐item scale rated on a 5‐point Likert scale for insomnia disorder screening. The SCI evaluates difficulty in initiating sleep, maintaining sleep, sleep quality, daytime sleep‐related symptoms, duration of sleep problems, nights per week having a sleep disturbance and extent troubled by poor sleep (Espie et al. 2014). Total scores range from 0 to 32, with lower scores indicating worse sleep. According to the recommendations of the authors of the original SCI version, insomnia disorder is defined by a total score of 16 or lower (Bayard et al. 2017). The structural invariance of the SCI has been established in different samples (Bayard et al. 2017, 2021).

### 2.3 Statistical Analysis

Most of the following analyses were conducted using JASP (JASP Team 2024). The receiver operator characteristic (ROC) analysis was performed with JAMOVI software [The jamovi project (2024). jamovi. (Version 2.6.23) retrieved from http://www.jamovi.org]. Means and standard deviations were computed for continuous variables, and categorical variables were expressed in percentages.

#### 2.3.1 Structural Invariance

Confirmatory factor analysis (CFA) was conducted in order to establish the structural invariance of the IHSS and the SCI (Kline 2023). The following indices were computed: *χ* ^*2*^ to degrees of freedom (*χ* ^*2*^ */df*, ≤ 3), the root mean square error of approximation (RMSEA ≤ 0.08), the comparative fit index (CFI > 0.90), normed fit index (NFI > 0.90) and the non‐normed fit index (NNFI > 0.90) (Byrne 2016). The analyses were conducted on the standardised variables. The internal consistency of the scales was examined with the omega coefficient. Values below 0.70 indicate poor reliability of the tool.

#### 2.3.2 Applicability of the SCI and IHSS Clinical Thresholds

The SCI total score was used to construct ROC curves. The area under the curve (AUC) was used to examine the SCI's accuracy in discriminating participants with insomnia disorder from those without insomnia. We derived cut‐points by plotting the relation between the sensitivity and 1 − specificity of the SCI total over all possible values on a ROC curve and selecting a clinical cutoff score that maximised both values. The further the ROC curve lies above a reference line, the more accurately a chosen cutoff score classifies positive and negative cases in a chosen sample (Mossman and Somoza 1991). Classification accuracy was evaluated using the following recommended ranges: low accuracy = AUC < 0.7, moderate accuracy = AUC between 0.7 and 0.9 and high accuracy = AUC > 0.9 (Swets 1988). For IHSS, we calculated the percentage of participants according to the threshold score of ≥ 22 and the severity categories proposed in the literature (Dauvilliers et al. 2019; Rassu et al. 2022).

#### 2.3.3 Network Analysis

Network analysis was conducted on SCI and IHSS items. The network consists of nodes (i.e., items), each representing a variable, which are connected by edges that typically signify correlational links. Partial correlation networks were estimated with the graphical Least Absolute Shrinkage and Selection Operator (gLASSO) (Friedman et al. 2008). The thickness of the edge indicates the strength of the correlation (thicker edges represent stronger correlations), with positive correlations shown in blue and negative correlations shown in red.

The centrality indices applied in the analysis included strength, closeness, betweenness and expected influence (Epskamp et al. 2018; Opsahl et al. 2010). The centrality indices computed in this study include strength, interdependence and closeness of nodes. Strength refers to the weighted sum and intensity of all connections associated with a specific node, indicating the node's overall influence within the network. A high strength centrality implies that a variable plays a crucial role in the network due to strong direct connections (without intermediaries) with other variables. Betweenness measures the number of shortest paths passing through a node, highlighting the node's role as an intermediary for information flow across different parts of the network. Closeness represents the extent of both direct and indirect connections between a specific node and all other nodes in the network. A variable with high closeness centrality rapidly impacts the entire network. The expected influence is a centrality metric in network analysis designed to quantify the overall influence of a node on the network. It extends the concept of strength centrality by incorporating the direction and valence (positive or negative) of connections. The extended Bayesian information criterion (EBIC) was used to select the optimal network model.

### 2.4 Procedure

The research protocol was carried out by two registered psychologists with expertise in the sleep field (C.M. and J.F.) and five trained final‐year master's students in psychology at the Univerty Montpellier Paul‐Valéry (France), under their supervision. Participants were university students recruited from January 2023 to March 2024 by means of advertisements, personal contacts and snowballing techniques (i.e., sampling technique, in which existing participants provide referrals to recruit others participants required for the study). They were met at the Univerty Montpellier Paul‐Valéry (France) (Epsylon Laboratory). Students were individually accommodated during a single session for about 45–60 min. They were informed about the nature and the objectives of the research, and they gave written consent. Participation was voluntary and that all data would remain confidential. Then the participants completed the clinical interview and questionnaires. Once the questionnaires had been completed, they were checked for missing data in the presence of the participant for possible completion. The study was carried out in accordance with the ethical principles of the Helsinki Declaration and the Ethics Committee of the local university (IRB ID: IRB00013307).

## 3 Results

### 3.1 Participant Characteristics and Descriptive Statistics of Study Measures

No participants were removed from the study prior to their completion either through self‐removal or investigator choice. Questionnaire and sociodemographic data are documented in Table 1.

**TABLE 1:** Sociodemographic characteristics and questionnaires of the total sample (*n* = 433).

Variables	Value	Skewness/Kurtosis
Socio‐demographics		
Age (years)	22 ± 2.4 [18–35]	
Gender (female, %)	52.7%	
Body mass index	22.7 ± 3.8 [15–40]	
Diploma (%)		
Psychology, social sciences and education	29.8%	
Biology, chemistry, math and physic	15.9%	
Marketing, economy and communication	12.5%	
Literature and arts	12%	
Law	8.3%	
Health sciences	6.7%	
Others	14.8%	
Part‐time job	28%	
Night shift	11.6%	
Sleep clinical interview		
Insomnia disorder	36.5%	
Restless legs syndrome/Willis–Ekbom	9.7%	
Obstructive sleep apnea risk	17.8%	
Questionnaires		
Idiopathic Hypersomnia Severity Scale (13‐item version)		
Total score	20.6 ± 8.1 [2–43]	0.05/−0.58
Nighttime sleep/inertia	8.7 ± 3.2 [1–17]	0.13/−0.54
Daytime consequences	7.5 ± 4.5 [0–19]	0.11/−0.52
Daytime sleepiness	4.4 ± 2.5 [0–11]	0.20/−0.23
Cut‐off score ≥ 22	71%	
Severity categories		
Mild (0–12)	17.1%	
Moderate (13–25)	52.3%	
Severe (26–38)	29.6%	
Very severe (39–50)	1.2%	
Sleep Condition Indicator		
Total score	19.4 ± 7.4 [2–32]	−0.11/−0.99
Nighttime symptoms	12.6 ± 5.4 [0–20]	−0.29/−0.29
Daytime symptoms	6.8 ± 3.04 [0–12]	−0.11/−0.78
Cut‐off score ≤ 13	23.8%	

*Note*: Mean ± standard deviation [range].

The sample consisted of 433 participants with an average age of 22.3 years (± 2.38), of whom 52.7% were female. Participants were enrolled in various bachelor's and master's programmes: 51% were bachelor's students (14.5% in the first year, 12.5% in the second year and 24% in the third year) and 49% were pursuing a master's degree. A third of them had a part‐time student job. Seventy‐three participants (36.5%) met clinical criteria for insomnia and 51 (10.9%) were on psychotropic medication, primarily antidepressants and anxiolytics. Additionally, 22% of the participants reported a chronic illness, with migraines, allergies and asthma being the most common conditions. Among the 433 participants, 9.8% were at risk of obstructive sleep apnea and 17.8% had RLS/WED symptoms. Among the participants at risk of obstructive sleep apnea, 73% had a positive score in Category 1 (snoring), 87% had a positive score in Category 2 (daytime sleepiness and fatigue) and 49% had a positive score in Category 3 (blood pressure/body mass index). None reported a self‐reported lifetime diagnosis of central disorders of hypersomnolence.

In our sample, one‐third of the participants were non‐drivers (*n* = 130). They were therefore unable to answer item 14 of the IHSS (*Do you consider your hypersomnolence to be an inconvenience for your driving?*). In view of this observation and to consider a sample as representative as possible of the French university population, the analyses described below concern the 13‐item IHSS version, not including item 14. The 14‐item IHSS analyses are nevertheless documented in the Supporting Information.

### 3.2 Structural Invariance

**FIGURE 1:**
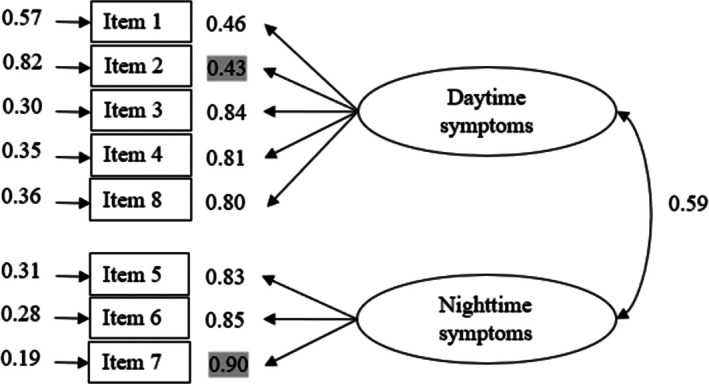
Path diagram with measurement error loadings and inter‐factor correlates for the Sleep Condition Indicator (*n* = 433). All manifest variables are represented by rectangles. Measurement errors (to the left of manisfest variables) and factor loadings (to the right of manisfest variables) are indicated by single‐head arrows. Minimum and maximum values of standardised loadings were highlighted.

For SCI, we tested a two‐dimensional model based on the results of the initial exploratory analysis of the scale within the general population (Bayard et al. 2017; Espie et al. 2014). The CFA was conducted using the diagonally weighted least squares method, considering the eight items of the scale. The model showed a good fit: *χ* ^*2*^_19_ = 44.3, *p* < 0.001; *χ* ^2^/df = 2.33; RMSEA = 0.06; CFI = 1; NFI = 0.99 and NNFI = 0.98. Figure 1 illustrates the SCI path diagram. The scale demonstrated a two‐factor structure composed of a first factor corresponding to the nighttime symptoms (i.e., Items 1, 2, 3, 4 and 8) and a second one referring to the daytime symptoms of insomnia (i.e., Items 5, 6 and 7). Omega coefficients suggest adequate internal consistencies for all SCI scores (total score, *ω* = 0.86; nighttime symptoms, *ω* = 0.84 and daytime symptoms factor, *ω* = 0.89).

**FIGURE 2:**
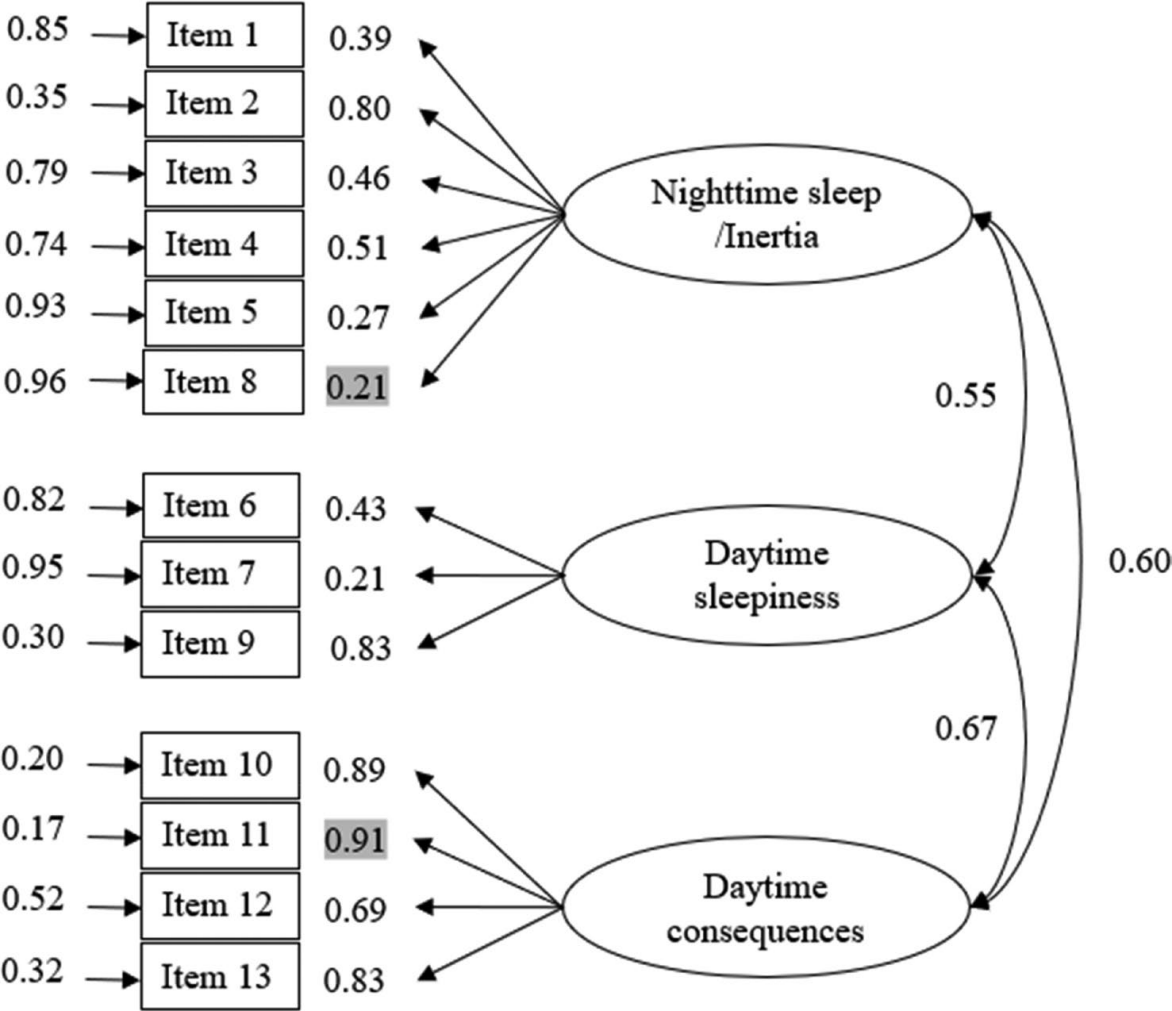
Path diagram with measurement error loadings and inter‐factor correlates for the 13‐item Idiopathic Hypersomnia Severity Scale (*n* = 433). All manifest variables are represented by rectangles. Measurement errors (to the left of manisfest variables) and factor loadings (to the right of manisfest variables) are indicated by single‐headedarrows. Minimum and maximum values of standardised loadings were highlighted.

A three‐dimensional model for the 13‐item IHSS, based on exploratory and confirmatory factor analyses in the general population, was tested (Madiouni et al. 2024). The CFA indicated a good fit: *χ* ^2^_62_ = 138, *p* < 0.001; *χ* ^2^/df = 2.22; RMSEA = 0.078; CFI = 0.91; NFI = 0.85 and NNFI = 0.89. The scale demonstrated a structure with three factors: the first, the nighttime sleep/inertia factor (Items 1, 2, 3, 4, 5 and 8); the second, the daytime sleepiness factor (Items 6, 7 and 8); and the third, the daytime consequences factor (Items 10, 11, 12 and 13) (Figure 2). Acceptable internal consistency was observed for the IHSS total score (*ω* = 0.84), daytime consequences (*ω* = 0.87), nighttime sleep/inertia (*ω* = 0.69) and daytime sleepiness factors (*ω* = 0.66). The model tested with the 14‐item IHSS version led to similar results (see Supporting Information).

### 3.3 Applicability of the SCI and IHSS Clinical Thresholds

ROC curve analyses showed that the total SCI score had the highest predictive value of insomnia disorder with a cutoff value of ≤ 13. The area under the ROC curve was 0.83 (75% sensitivity and 93% specificity). Regarding the cutoff value of ≤ 16 established in the general population (Bayard et al. 2017; Espie et al. 2014), the area under the ROC curve was 0.78 (60% sensitivity and 96% specificity). With regard to IHSS, 71% of the participants scored at or above the threshold for the idiopathic hypersomnia screening (i.e., ≥ 22). Regarding the severity categories, 81.9% of the participants reported moderate to severe symptoms (Table 1).

### 3.4 Network Analysis

#### 3.4.1 Centrality Indices

The sparsity value was 0.63, suggesting that the network is relatively sparse, with limited interconnectivity among nodes (symptoms), which may point to specific clusters or dependencies on key nodes.

**FIGURE 3:**
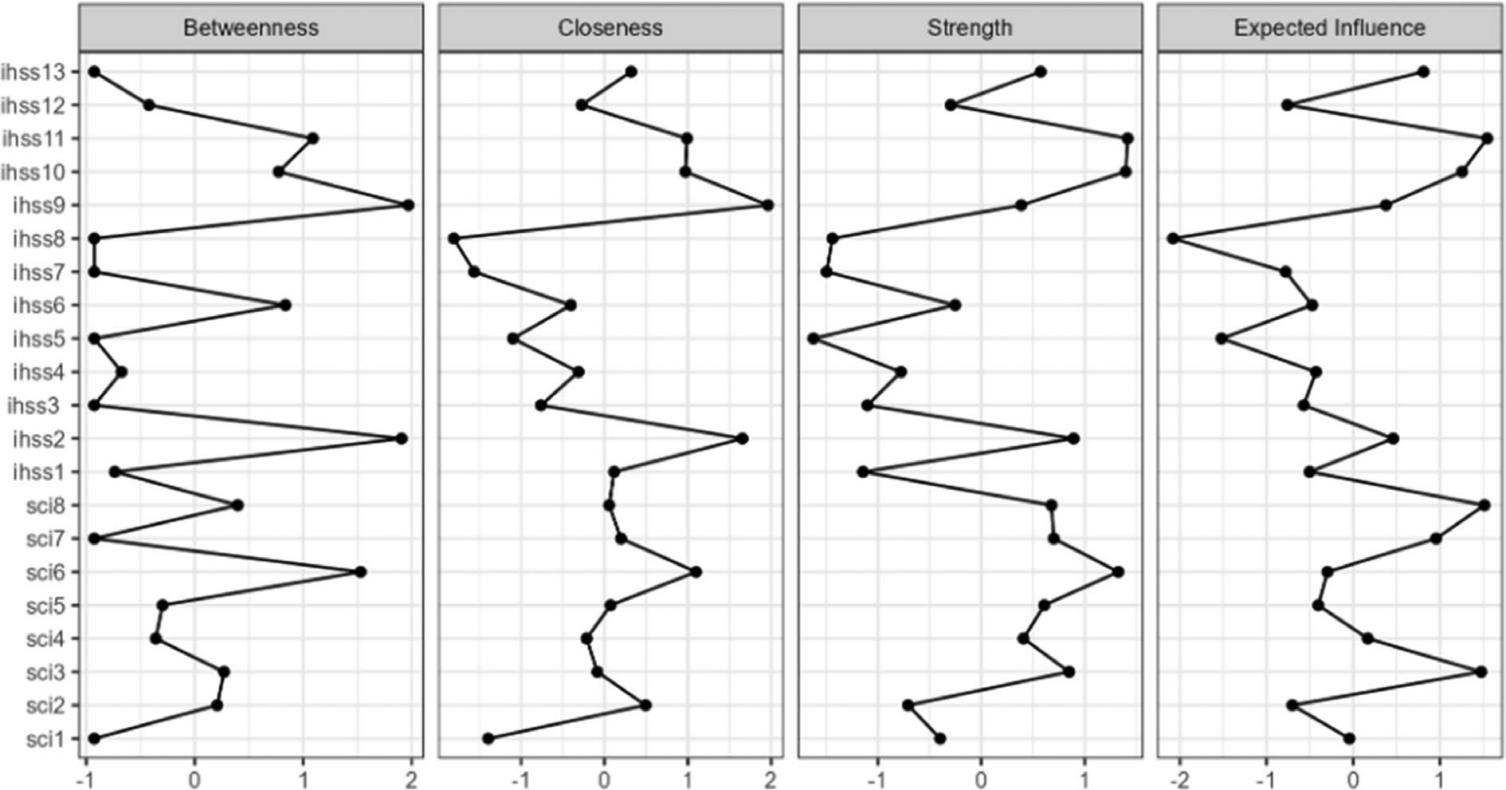
Centrality plot depicting standardised centrality indices (betweenness, closeness and strength) and the SCI and IHSS networks.

The central indices for all nodes in the network are shown in Figure 3. In terms of local structure, the three most central symptoms playing a bridge role in the network graphical representation, with the highest strength were: IHSS items 10 ‘impact on general health (i.e., lack of energy, no motivation to do things, physical fatigue on exertion, decrease in physical fitness)’ (*z* = 1.39); IHSS item 11 ‘problem in terms of intellectual function’ (*z* = 1.41) and SCI 6 item ‘impact on concentration, productivity, or ability to stay awake’ (z = 1.32). This means that these symptoms exhibit a high degree of connection in the entire network (strength). Note that, in our sample, these two IHSS items were collinear (*r* = 0.82).

The three symptoms with the highest betweenness and closeness values were IHSS Item 9 ‘struggling to stay awake’ (respectively, *z* = 1.97; *z* = 1.96); IHSS Item 2 ‘sensation of not having slept enough when circumstances require waking up at a particular time in the morning’ (respectively, *z* = 1.91; closeness: *z* = 1.65) and SCI Item 6 ‘impact on concentration, productivity, or ability to stay awake’ (respectively, *z* = 1.52; *z* = 1.10). High closeness indicates that a symptom is efficiently connected to all other symptoms (low average shortest path), while high betweenness signifies that a symptom frequently acts as a bridge or intermediary in the shortest paths between other symptoms.

Finally, the IHSS item 10/11, the SCI Item 3 ‘weekly frequency of sleep problems’ and SCI Item 8 ‘sleep problems duration’ had the highest expected influence within the network (respectively, *z* = 2.65; *z* = 2.96; *z* = 2.33; *z* = 2.19). This centrality index allows for quantifying the overall influence of a symptoms within the network.

#### 3.4.2 Interrelationships Between Insomnia and Hypersomnolence Symptoms

**FIGURE 4:**
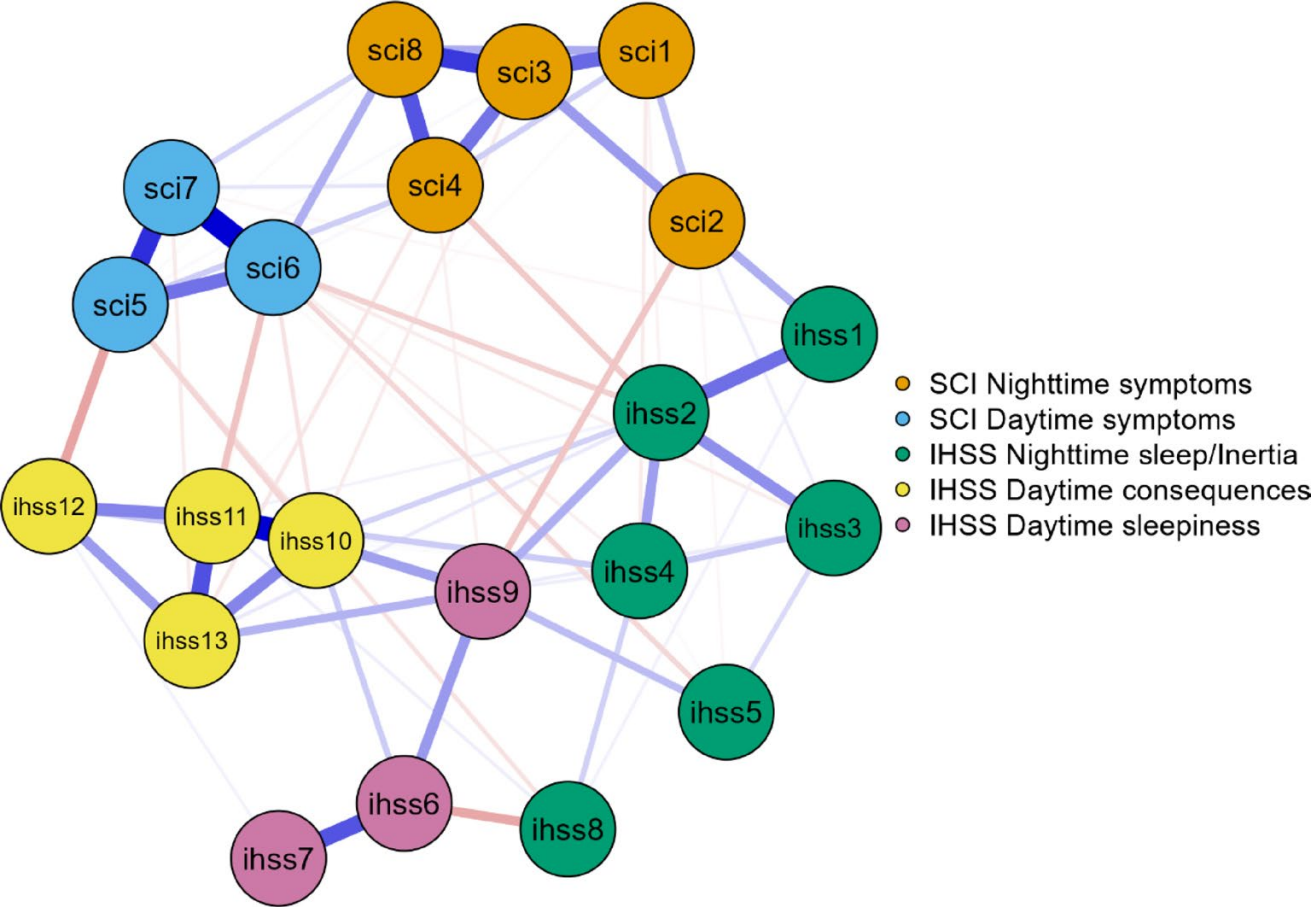
Estimated EBIC gLASSO network of insomnia and hypersomnolence symptoms (nodes). The blue edges (links) represent the positive associations between symptoms and the red edges the negative associations. IHSS, Idiopathic Hypersomnia Severity Scale; SCI, Sleep Condition Indicator.

Interestingly, five main clusters can be easily visually identified, confirming the factorial organisation of the SCI and the IHSS. Two clusters of nodes represent the daytime and nighttime symptoms of insomnia assessed by the SCI; three others represent the three dimensions of hypersomnolence from the IHSS (i.e., daytime consequences; nighttime sleep/inertia; daytime sleepiness) (Figure 4).

A detailed analysis of the relationship between insomnia and hypersomnolence symptoms revealed a moderate interconnection between their cognitive consequences (SCI Item 6 ‘impact on concentration, productivity, or ability to stay awake’/IHSS Item 11 ‘problem in terms of intellectual function’).

Higher cognitive complaints related to insomnia (SCI Item 6) were associated with increased feelings of insufficient sleep and more pronounced sleep inertia, as well as a greater complaints about the consequences of hypersomnolence on general health (respectively, IHSS Item 2 ‘sensation of not having slept enough when circumstances require waking up at a particular time in the morning’; IHSS Item 5 ‘doing irrational things and/or say irrational things, and/or are you very clumsy’; IHSS Item 10 ‘impact on general health (i.e., lack of energy, no motivation to do things, physical fatigue on exertion, decrease in physical fitness)’).

These interrelations were, however, of a more modest scale. However, they have led us to conduct an exploratory mediation analysis to test the mediating role of sleep inertia (IHSS Item 5) in the association between feelings of insufficient sleep (IHSS Item 2) and cognitive complaints related to insomnia (SCI Item 6) (Hayes 2022). Regarding the component effects, feelings of insufficient sleep predicted sleep inertia (*β* = 0.25, *p* < 0.001); sleep inertia predicted insomnia cognitive complaints (*β* = −0.17, *p* < 0.001). A mediating effect of feelings of insufficient sleep on insomnia cognitive complaints via sleep inertia was observed (*β* = −0.04, *p* < 0.01). Total and direct effects were also significant (respectively, *β* = −0.39, *p* < 0.001; *β* = −0.35, *p* < 0.001) (Table 2).

**TABLE 2:** Indirect and total effects of the mediating effect of sleep inertia (IHSS 5) on the relationship between insufficient sleep (IHSS 2) on insomnia cognitive complaints (SCI 6).

				95% confidence intervals		*β*	*z*	*p*
Type	Effect	Estimate	SE	Lower	Upper			
Indirect	IHSS 2 ⇒ IHSS 5 ⇒ SCI 6	−0.06	0.02	−0.09	−0.02	−0.04	−3.09	< 0.01
Component	IHSS 2 ⇒ IHSS 5	0.24	0.04	0.15	0.32	0.25	5.40	< 0.001
	IHSS 5 ⇒ SCI 6	−0.24	0.06	−0.36	−0.11	−0.17	−3.76	< 0.001
Direct	IHSS 2 ⇒ SCI 6	−0.47	0.06	−0.58	−0.34	−0.35	−7.69	< 0.001
Total	IHSS 2 ⇒ SCI 6	−0.52	0.06	−0.63	−0.40	−0.39	−8.76	< 0.001

Abbreviations: IHSS = Idiopathic Hypersomnia Severity Scale; SCI = Sleep Condition Indicator; SE = standard error.

Affective symptoms of insomnia and hypersomnolence were moderately interconnected (SCI Item 5 ‘effect on mood, energy, or relationships’/IHSS Item 12 ‘hypersomnolence effect on mood [e.g., sadness, anxiety, hypersensitivity, irritability]’). Higher level of affective insomnia symptoms was weakly associated to a greater complaint about the consequences of hypersomnolence on general health (IHSS item 10 ‘impact on general health (i.e., lack of energy, no motivation to do things, physical fatigue on exertion, decrease in physical fitness)’).

Additionally, greater wakefulness after sleep onset (SCI Item 2) was linked to both a shorter ideal night‐time sleep duration and increased difficulty staying awake during low‐stimulation activities throughout the day (respectively, IHSS Item 1 ‘ideal duration of night‐time sleep, at the weekend or on holiday, for example’; IHSS Item 9 ‘struggling to stay awake while carrying out activities that are not very stimulating during the day’).

## 4 Discussion

The results of this research provide supporting evidence that the SCI and IHSS have satisfactory structural invariance in the sample of university students recruited. A SCI cutoff score of ≤ 13 appears appropriate for the diagnosis of insomnia in this population. More than two‐thirds of them had a score above the clinical threshold of the IHSS (i.e., ≥ 22). With regard to network analysis applied to SCI and IHSS items, the consequences of hypersomnolence symptoms on general health and intellectual functioning, as well as the cognitive consequences of insomnia, were the symptoms with the highest strength of centrality and expected influence. Daytime sleepiness, insufficient sleep and insomnia cognitive complaint were the symptoms with the highest strength of closeness and betweenness. The consequences of hypersomnolence symptoms on general health and intellectual functioning, as well as the frequency and duration of sleep problems, had the highest expected influence with the network. Cognitive and affective symptoms of insomnia and hypersomnolence were interconnected. Greater cognitive complaints related to insomnia were linked to heightened feelings of insufficient sleep, more noticeable sleep inertia and increased concerns about the impact of hypersomnolence on overall health. Finally, increased wakefulness after falling asleep was associated with both a shorter preferred night‐time sleep duration and greater difficulty staying alert during low‐stimulation activities throughout the day. Since IHSS Items 10 and 11 were collinear, they will be discussed together. Notably, certain clinical aspects of IHSS Item 10 are theoretically underpinned by cognitive factors, particularly energy, motivation and fatigue (Hockey 2013).

In a sample of university students, we established the structural stability of the SCI. However, the previously established threshold score of ≤ 16 for the general population (Bayard et al. 2017) was not suitable for identifying insomnia in students; it has been revised to ≤ 13. Notably, the prevalence of insomnia disorder based on international classifications in university students is unknown. Our results showed that 36.5% of students met the criteria for insomnia disorder, which is three times higher than in the general population and our initial validation sample (Bayard et al. 2017; Morin and Jarrin 2022). In this high‐prevalence context, even a moderate insomnia complaint score may indicate a more serious clinical risk. CFA of the IHSS confirmed the validity of its three‐factor structure (Madiouni et al. 2024). In our sample, 71% of students scored above the IHSS threshold of ≥ 22, the optimal cutoff for differentiating untreated idiopathic hypersomnia patients from controls and patients with Type 1 narcolepsy. This high proportion is surprising, given that idiopathic hypersomnia is considered rare, with a prevalence of 10.3 per 100,000 people (Acquavella et al. 2020). Concerning the IHSS severity categories, 81.9% of the students indicated experiencing moderate to severe symptoms. Consequently, we question the appropriateness of applying this threshold to a student population, as the IHSS may lack specificity in this context. Establishing clinical thresholds that are both specific and sensitive to the population of interest is essential. However, many such thresholds, including for insomnia and sleepiness, are still lacking or inconsistently applied in university settings (Babicki et al. 2023; Dagnew et al. 2020; Dietch et al. 2016; Da Dutra Silva et al. 2022; Emert et al. 2024; Ramos et al. 2021). It should be noted that the risk of obstructive sleep apnea and the frequency of RLS/WED observed in our sample are in line with literature data (Ohayon et al. 2012; Zasadzińska‐Stempniak et al. 2024).

Centrality indices, particularly, strength and expected influence, highlighted that the impact of hypersomnolence on intellectual functioning, the cognitive effects of insomnia and its chronicity were the most significant nodes within the network, exerting a notable overall influence. We also observed an association between cognitive symptoms of insomnia and hypersomnolence. As noted above, university students are particularly vulnerable to sleep–wake disturbances. In the context of their studies, they rely heavily on cognitive functions, especially executive functions, which are critical not only for academic success but also for adapting to the demands of their environment. Our findings align with extensive literature highlighting the pivotal role of intellectual functioning, particularly, executive functions, in academic achievement across the lifespan (Best et al. 2011). Moreover, they are consistent with evidence that sleep–wake disturbances impair cognitive abilities, especially executive functions (Brownlow et al. 2020; Filardi et al. 2021). While research on university students remains limited, several studies indicate that insufficient sleep and sleepiness are linked to poor executive functioning in this population (Albuquerque et al. 2023; Parrilla et al. 2024).

In addition to the relationships found between the cognitive consequences of insomnia and hypersomnolence, we also noted links between their emotional consequences, more specifically on mood. From their very first year at university, students represent a population at risk of developing psychopathological conditions, of which mood disorders are among the most representative (Duffy et al. 2020). There is also some evidence that symptoms of insomnia and daytime sleepiness are closely associated with depressive symptoms in this population (Ramos et al. 2021; Seehuus et al. 2024). In addition, supporting and encouraging healthy sleep among university students improves their mental health, particularly, by reducing depressive and anxiety symptoms (Chandler et al. 2022).

The present study found a relationship between difficulties maintaining sleep and alertness during low‐stimulation activities throughout the day. These two symptoms acted as key links in the symptom network, with high betweenness and closeness, meaning they both connected other symptoms and could influence or be influenced by the entire network. While a few exceptions exist (Babicki et al. 2023; Carrión‐Pantoja et al. 2022), most studies linking nighttime sleep and daytime sleepiness in university students have used clinical thresholds from the PSQI and ESS (Da Dutra Silva et al. 2022; Jahrami et al. 2019; Ramos et al. 2021). These studies suggest that poor sleep quality, as measured by the PSQI, predicts clinically significant excessive daytime sleepiness (ESS ≥ 10), a threshold that has not been validated in this population. Babicki and colleagues found a small positive association between the Athens Insomnia Scale and ESS in a sample of Polish students, most of whom were women (i.e., 80.7%) (Babicki et al. 2022). However, these studies failed to pinpoint which specific nocturnal insomnia symptoms contribute to daytime alertness problems. Nighttime issues in insomnia, such as difficulty initiating or maintaining sleep, nonrestorative sleep or a combination, may independently influence daytime symptoms (Roth et al. 2010). Our findings align with the Brazilian Epidemiological Sleep Study, which documented a relationship between ESS scores and the polysomnographic wake‐after‐sleep‐onset index, also linked to self‐reported sleep duration (Fernandes et al. 2023).

We also reported that greater wakefulness after sleep onset was related to shorter preferred night‐time sleep duration. This observation is consistent with polysomnographic studies documenting that sleep fragmentation can influence the perception of ideal sleep duration, as non‐restorative sleep can lead to inaccurate judgements about one's needs (Benkirane et al. 2024). Students with increased intra‐sleep wakefulness may perceive their sleep as being of poorer quality, leading them to believe they need fewer hours of sleep to ‘feel good’ or to underestimate their actual sleep requirements.

Sleep inertia is often overlooked in university settings. One study suggests that snooze alarms may worsen sleep inertia through repeated forced awakenings, while another indicates that self‐awakening can reduce its effects (Ikeda and Hayashi 2010; Ogawa et al. 2022). In our sample, the IHSS item related to sleep drunkenness (IHSS Item 5) was linked to greater cognitive complaints associated with insomnia, which were in turn connected to feelings of insufficient sleep. This supports previous research that inertia is most intense during awakenings and impacts cognition (Hilditch and McHill 2019; Ogawa et al. 2022; Trotti 2017). A mediation analysis revealed that sleep inertia played a mediating role between insufficient sleep and cognitive complaints. Descriptive analyses showed that 40% of students found it extremely difficult or impossible to wake without multiple alarms or assistance (IHSS Item 3) and 15% reported symptoms of sleep drunkenness (IHSS Item 5). These findings emphasise the need to address sleep inertia in university students with hypersomnolence.

This study has several limitations that should be considered when evaluating its findings. First, the study was conducted exclusively on French university students, which limits the generalisability of the findings to broader populations, such as students from other countries or individuals outside the academic environment. Second, the study's cross‐sectional design precludes conclusions about causal relationships between insomnia and hypersomnolence symptoms. Third, we used the Berlin Questionnaire, which did not allow us to screen for the risk of central or complex sleep apnea. However, none of the participants in our study reported chronic illnesses such as heart failure, which are associated with these sleep‐related breathing disorders, in particular, Cheyne‐Stokes breathing (Roberts et al. 2022). Cerebrovascular diseases, including stroke and other medical conditions associated with central apnea were listed as exclusion criteria. Our sample had an average age of 22 years. Younger age is a protective factor for central or complex sleep apnea. Finally, our sample size was modest. Although network analyses are relatively recent and lack a consensus on the calculation of the required sample size, this exploratory network study is the first to investigate the interrelationships between insomnia and hypersomnolence symptoms as defined by international classifications.

This study examined the interrelationships between insomnia and hypersomnolence symptoms among French university students, introducing a novel network analysis approach. It confirms the structural invariance of both the SCI and IHSS in this population. In our sample, more than a third of students met the criteria for insomnia disorder, making it the most prevalent psychopathological condition. Additionally, we observed a notably high frequency of hypersomnolence symptoms, including sleep inertia, a phenomenon not previously described in this group. The innovative approach of this study underscores the intricate relationship between insomnia and hypersomnolence, highlighting the urgent need for targeted interventions that address both daytime and nighttime sleep–wake disturbances. In this regard, it is unfortunate that French health authorities have yet to prioritise sleep in their health prevention recommendations for university students (https://www.etudiant.gouv.fr/fr/la‐sante‐sur‐les‐campus‐910).

## Author Contributions

**Sophie Bayard:** conceptualization, methodology, data curation, validation, formal analysis, supervision, funding acquisition, visualization, project administration, resources, writing – original draft. **Julie Faccini:** validation, visualization, writing – review and editing, resources. **Jonathan Del‐Monte:** validation, visualization, writing – review and editing, resources. **Clarisse Madiouni:** conceptualization, investigation, writing – review and editing, data curation, methodology, validation, visualization, formal analysis.

## Conflicts of Interest

The authors declare no conflicts of interest.

## Supporting information


**Data S1.** Supporting Information.

## Data Availability

The data that support the findings of this study are openly available in DOI 10.17605/OSF.IO/D7B64 at https://osf.io/d7b64/.
