# Supplementary material for: The Sleep Condition Indicator and the Idiopathic Hypersomnia Severity Scale: Measurement Invariance and an Exploratory Network Approach in a French Sample of University Students
Source: J Sleep Res. 2025 Apr 10;34(5):e70042. doi: 10.1111/jsr.70042 (PMC12426702; doi:10.1111/jsr.70042)

**Structural invariance**

A three-dimensional model for the 14-item IHSS, based on exploratory and confirmatory factor analyses in the general population, was tested (Madiouni et al., 2024). The CFA indicated a good fit: *χ*^2^_74_ = 156, *p* < 0.001; *χ*^2^/df = 2.10; RMSEA = 0.074; CFI = 0.91; NFI = 0.84 and NNFI = 0.89. The scale demonstrated a structure with three components: the first, the Nighttime Sleep/Inertia factor (items 1, 2, 3, 4, 5, and 8); the second, the Daytime Sleepiness factor (items 6, 7, and 8); and the third component (items 10, 11, 12, 13, and 14) (Figure 1). Acceptable internal consistency was observed for the IHSS Total score (*ω* = 0.85), Daytime consequences (*ω* = 0.87), Nighttime sleep/Inertia (*ω* = 0.69) and Daytime sleepiness factors (*ω* = 0.68).

Figure 1. Path diagram with measurement errors loadings and inter-factors correlates for the 14-item Idiopathy Hypersomnia Severity Scale (*n* = 433). All manifest variables are represented by rectangles. Measurement errors (to the left of manisfest variables) and factor loadings (to the right of manisfest variables) are indicated by single-head-arrows. Minimum and maximum values of standardized loadings were highlighted.


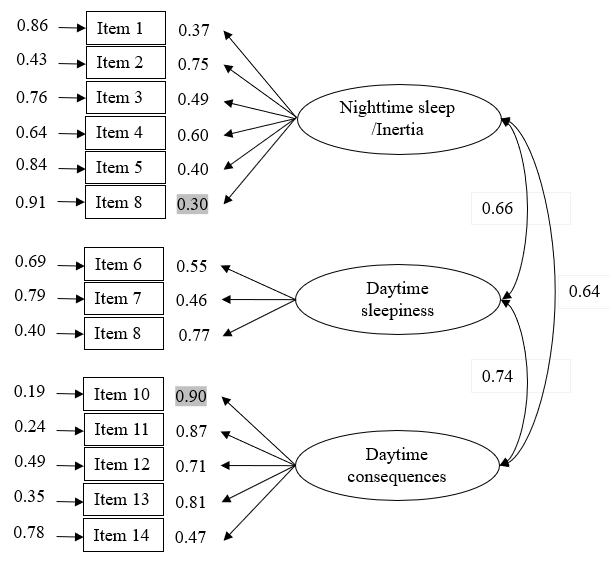


**Network analysis**

The sparsity value was 0.62, suggesting that the network is relatively sparse, with limited interconnectivity among nodes (symptoms), which may point to specific clusters or dependencies on key nodes.

The central indices for all nodes in the network are shown in Figure 2. In terms of local structure, the three most central symptoms playing a bridge role in the network graphical representation, with the highest strength were: IHSS items 10 “*impact on general health (i.e., lack of energy, no motivation to do things, physical fatigue on exertion, decrease in physical fitness)*” (z = 1.37); IHSS item 11 “*problem in terms of intellectual function*” (z = 1.64); and SCI 6 item “*impact on concentration, productivity, or ability to stay awake*” (z = 1.30). This mean that these symptoms exhibit a high degree on connection in the entire network (strength).

The three symptoms with the highest betweenness and closeness values were: IHSS item 9 “*struggling to stay awake*” (respectively, z = 2.30; z = 1.63); IHSS item 2 “*sensation of not having slept enough when circumstances require waking up at a particular time in the morning*” (respectively, z = 1.52; closeness: z = 1.44); and SCI item 6 “*impact on concentration, productivity, or ability to stay awake*” (respectively, z = 1.47; z = 1.22). High closeness indicates that a symptom is efficiently connected to all other symptoms (low average shortest path), while high betweenness signifies that a symptom frequently acts as a bridge or intermediary in the shortest paths between other symptoms.

Finally, the IHSS item 10/11, the SCI item 3 “*weekly frequency of sleep problems”* and SCI item 8 “*sleep problems duration”* had the highest expected influence within the network (respectively, z = 1.21; z = 1.85; z = 2.33; z = 1.51).

Interestingly, five main clusters can be easily visually identified confirming the factorial organization of the SCI and the IHSS. Two clusters of nodes represent the daytime and nighttime symptoms of insomnia assessed by the SCI; three others represent the three dimensions of hypersomnolence from the IHSS (i.e., Daytime consequences; Nighttime sleep/Inertia; Daytime sleepiness) (Figure 3).

Figure 2. Centrality plot depicting standardized centrality indices (betweenness, closeness, and strength) and the SCI and 14-item IHSS version network.


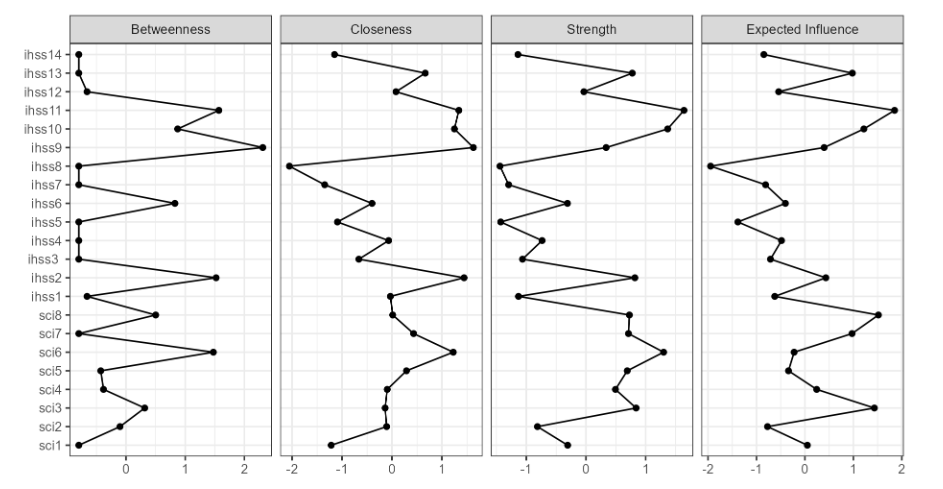


Figure 3. Estimated EBIC gLASSO network of insomnia and hypersomnolence symptoms (nodes). The blue edges (links) represent the positive associations between symptoms and the red edges the negative associations. IHSS: 14-items Idiopathic Hypersomnia Severity Scale version; SCI: Sleep Condition Indicator.


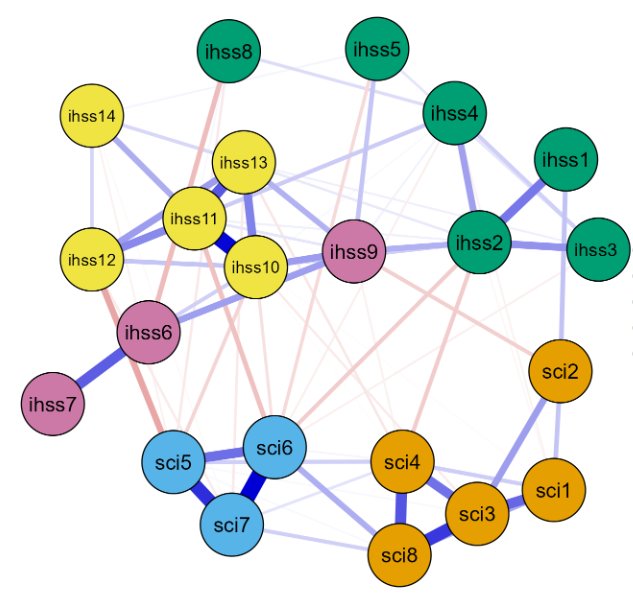


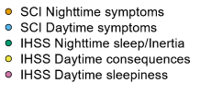

Supplement: Supplementary file 1 — Data S1. Supporting Information. [file JSR-34-e70042-s001.docx]
